# Association between the combination of GABAergic agents and SSRIs at the first clinical visit and depressive symptom trajectories: A study using group-based trajectory modeling and Apriori algorithm

**DOI:** 10.1371/journal.pmen.0000544

**Published:** 2026-07-14

**Authors:** Bei Tang, Lu Jin, Hongxing Hu, Qinyu Lv, Zhenghui Yi, Yuelong Ji, Qizhong Yi

**Affiliations:** 1 Department of Maternal and Child Health, School of Public Health, Peking University, Beijing, China; 2 Shanghai Mental Health Center, Shanghai Jiao Tong University School of Medicine, Shanghai, China; 3 Psychological Medicine Center, The First Affiliated Hospital of Xinjiang Medical University, Urumqi, Xinjiang, China; 4 Xinjiang Clinical Research Center for Mental Health, Urumqi, China; 5 Department of Psychiatry, Huashan Hospital, Fudan University, School of Medicine, No. 12 Wulumuqi Road (middle), Shanghai, China; 6 Institute of Mental Health, Fudan University, Shanghai, China; PLOS: Public Library of Science, UNITED KINGDOM OF GREAT BRITAIN AND NORTHERN IRELAND

## Abstract

Depression is a leading cause of disability, with limited efficacy of current treatments: 30% - 40% of major depressive disorder patients fail first-line therapy, and relapse rates remain high. Combination pharmacotherapy shows promise, but real-world evidence on initial treatment strategies and longitudinal symptom trajectories is lacking. We conducted a longitudinal analysis of electronic health records from 1,876 patients who visited the Shanghai Mental Health Center between November 2021 and April 2023. Group-based trajectory modeling (GBTM) classified patients into subgroups based on PHQ-9 score trajectories. The Apriori algorithm identified common medication mechanism combinations for the first visit. Multinomial logistic regression evaluated associations between medication groups and trajectory subgroups. General linear modeling examined SDS score improvement. Network pharmacology was conducted as an exploratory analysis to investigate potential biological context. Three PHQ-9 trajectory groups were identified: Rapid Decline (29.96%), Gradual Decline (30.76%), and Worsening (39.28%). The Worsening Group had lower initial PHQ-9 scores but showed progressive severity elevation. Four medication mechanism combination groups were identified including GABAergic agents alone (GABA), selective serotonin reuptake inhibitors alone (SSRIs), GABA+SSRIs combination, and Others. GABA+SSRIs was the most frequent combination (31.02%). Compared with GABA+SSRIs, patients in the SSRIs group (OR _adj_, 1.809; 95% CI, 1.196 - 2.736) had significantly higher odds of belonging to the Worsening Group (*P* = 0.005). In the supplementary SDS analysis, GABA monotherapy was associated with significantly less symptom improvement than GABA+SSRIs (β = -1.790, 95% CI: -3.320, -0.258). Network analysis identified 33 intersecting targets between GABA+SSRIs and depression, enriched in neurotransmission and synaptic plasticity pathways. Overall, the combination of GABAergic agents and SSRIs at the first clinical visit was associated with a more favorable PHQ-9 trajectory group than SSRIs monotherapy. These findings may inform future prospective and mechanistic studies on medication mechanism combinations in depression treatment.

## Introduction

Depression ranks as the second leading cause of disability within non-communicable diseases on a global scale. In 2021, there were around 332 million cases globally, and this number is continuing to climb [1–4]. Current treatments typically include antidepressants (e.g., selective serotonin reuptake inhibitors [SSRIs]), psychotherapy, and other biological interventions etc [5]. But numerous studies have demonstrated that existing antidepressant therapies show limited efficacy, with 30% to 40% major depressive disorder (MDD) patients failing to first-line therapy, alongside high rates of relapse [6–12]. Antidepressants also have many drawbacks, including delayed onset of action, single-target mechanisms, and multiple adverse effects [13–16]. Many patients still require a trial-and-error approach to medication adjustment [17]. These limitations underscore the urgent need for innovative pharmacological strategies for depression.

Clinical pharmacotherapy for depression currently lacks clear guidelines, and prescribing patterns are often influenced by physician preference and experience. More beneficial medication approaches need to be found. Recent studies show that combination therapy outperforms monotherapy, showing improved efficacy and lower treatment discontinuation rates [18–23]. Similarly, augmenting antidepressants with other medications, including thyroid hormones and herbal preparations, can result in significantly better outcomes than monotherapy [24,25]. But clinical application is often hampered by methodological limitations: many studies fail to account for heterogeneous longitudinal symptom trajectories and recognize complex real-world patterns of polypharmacy.

Latest advances in statistical methods have created avenues for mental health research. Group-based trajectory modeling (GBTM), a longitudinal data analysis technique, is increasingly utilized in psychiatric research to identify subgroups and reveal distinct developmental trajectories [26–28]. The Apriori algorithm are also being applied to explore frequent itemsets in psychiatric treatments, revealing hidden patterns in therapeutic practices [29–32]. These methods offer an innovative approach to studying heterogeneity in psychopharmacology, moving beyond traditional hypothesis-driven research.

This research represents a novel application of machine-learning techniques within the realm of psychiatric pharmacotherapy, by harnessing the complementary strengths of the GBTM and Apriori algorithms. Our aim was two-fold: first, to examine the associations between particular mechanism combinations and trajectory groups; second, to explore initial clinical visit medication combinations associated with more favorable depression rating scales outcomes. Uniquely, we also centered our investigation on medication mechanism combinations, thereby laying the groundwork for formulating prescriptions during the first clinical encounter. This approach enriches the methodological toolkit in psychiatric research and holds significant potential for clinical practice in treating depression.

## Materials and methods

### Ethics statement

This study was performed in line with the principles of the Declaration of Helsinki. Approval was granted by the Ethics Committee of Shanghai Mental Health Center with approval no. 2023–62.

For patient data use, we employed an opt-out informed consent mechanism approved by the Ethics Committee. All potential participants, upon registration or clinical visit, received a comprehensive information letter detailing the study’s purpose, procedures, risks, benefits, and confidentiality measures, including the potential use of their records for research. The letter explicitly stated that participation was voluntary and that by not contacting the research team via the provided means within two weeks, they provided their consent to participate. No participants opted out.

All data used in the analysis were de-identified and kept confidential prior to researcher access to protect patient privacy.

### Study design and workflow

This study was conducted in a series of steps, following the workflow shown in S1 Fig.

Briefly, GBTM was used to identify longitudinal PHQ-9 trajectory subgroups; frequent itemset mining was applied to baseline medication mechanisms to derive medication mechanism combination groups; multinomial logistic regression was used to evaluate associations between medication mechanism combination groups and PHQ-9 trajectory subgroups; and network pharmacological analysis was conducted as an exploratory approach to investigate potential biological mechanisms underlying the observed associations.

### Study population

This was a retrospective longitudinal study utilizing electronic health records (EHR) data from the outpatient clinic of the Shanghai Mental Health Center. The study cohort included 2975 patients who visited the clinic between 10/11/2021 and 06/04/2023, received pharmacological treatment and completed depressive symptom assessments during the study period. Eligible patients were required to have: (1) documented pharmacological treatment information; (2) at least two PHQ-9 measurements, including one baseline and at least one follow-up assessment; and (3) baseline and final SDS assessments.

Data were prospectively collected during routine clinical visits through multiple mental health and physical health-related questionnaires. Every patient is assigned a unique personal identification number upon registration, which enabled the exact individual-level linkage of the longitudinal data from the outpatient visits. Pharmacological treatment information was extracted from EHR medication records. Psychotherapy information and treatment duration before inclusion were not consistently available in the structured outpatient EHR data and were therefore not included as covariates. The first available PHQ-9 assessment during the study period was defined as the baseline assessment. The final dataset for this specific retrospective analysis was accessed on 20/08/2024.

### Patient Health Questionnaire-9 (PHQ-9)

PHQ-9 was used to assess depressive symptom severity during routine outpatient visits. The PHQ-9 assesses symptoms experienced over the preceding two weeks and consists of 9 items, with each item scored from 0 to 3 [33]. The total PHQ-9 score was calculated by summing the scores of all 9 items, yielding a total score ranging from 0 to 27, with higher scores indicating greater depressive symptom severity. Cutoff scores of 5, 10, 15, and 20 indicate mild, moderate, moderately severe, and severe depressive symptoms, respectively. In this study, PHQ-9 was not administered daily; rather, it was completed during outpatient visits as part of routine clinical assessment. Therefore, the number and timing of PHQ-9 measurements varied across patients. Repeated PHQ-9 measurements were used for longitudinal modeling of depressive symptom trajectories. The reliability and validity of PHQ-9 have been examined in the Chinese [34,35].

### Zung’s Self-Rating Depression Scale (SDS)

SDS was used as an additional self-report measure of depressive symptoms during routine outpatient visits. The SDS consists of 20 items covering psychological and physiological symptoms, with each item scored from 1 to 4 [36]. The raw SDS score was calculated by summing the 20 item scores, yielding a total score ranging from 20 to 80. The raw score was converted to a standard score by multiplying by 1.25, yielding a standard score ranging from 25 to 100, with higher scores indicating more severe depressive symptoms. A standard score of 50 is commonly used as the cutoff for clinically significant depressive symptoms. In this study, SDS was measured at baseline and at the final available assessment. The difference between the baseline and final SDS standard scores was calculated to evaluate symptom improvement in the secondary analysis. Because SDS reflects symptoms over a recent recall period, the difference between baseline and final SDS standard scores was interpreted as a supplementary and coarse measure of endpoint symptom change rather than as a continuous assessment of symptom fluctuations during follow-up. The reliability and validity of SDS have been examined in the Chinese [37].

### Medication mechanism combinations

Medication information is obtained from outpatient records. Since medications were recorded under multiple trade names, all medications were mapped to their corresponding pharmacological mechanism based on pharmacological principles and authoritative standards for medication classification, i.e., DrugBank (https://www.drugbank.ca/) [38]. The medication mechanisms prescribed at the first clinical visit were used to define each patient’s baseline medication mechanism profile. The first-visit medication mechanism was subsequently used to assign patients to medication mechanism combination groups. We focused on the first-visit prescription because initial treatment selection represents an important clinical decision point that may influence subsequent symptom trajectories, and because treatment regimens frequently change over time in outpatient settings.

### Trajectory groups

GBTM was used to identify latent subgroups of patients with similar longitudinal PHQ-9 symptom trajectories after the baseline visit, which served as the primary outcome categories in the subsequent association analyses [39]. In the R 4.4.2 environment, we employed the “lcmm” package to fit trajectory models with 2–7 groups. For each group number, linear, quadratic, and cubic polynomial models were applied. The optimal model was selected based on a comprehensive comparison of model fit indices and classification quality metrics, including the Akaike Information Criterion (AIC), Bayesian Information Criterion (BIC), sample-size adjusted BIC (ABIC), average posterior probability of assignment (APPA/AvePP), odds of correct classification (OCC), and entropy, together with assessment of trajectory morphology and clinical interpretability (S1 Table). AvePP values above 0.70 and OCC values above 5.0 were considered indicators of acceptable classification quality. The selected model was a 3-group cubic model. Each patient was assigned to the trajectory group with the highest posterior probability.

### Frequent itemset mining

Frequent Itemset Mining (FIM) was used to identify common baseline medication mechanism combinations [40,41]. In this analysis, each patient’s first-visit prescription was treated as one record, and the pharmacological mechanisms of medications prescribed at that visit were treated as the elements within that record. A frequent itemset therefore referred to a medication mechanism combination that appeared repeatedly across patients. We used MLxtend for Python 3.12.7 to identify frequent medication mechanism itemsets. The Frequent Pattern Growth (FP-Growth) algorithm was used to construct Frequent Pattern (FP) trees, which summarized and visualized the co-occurrence structure of medication mechanisms (min count = 6, max depth = 5) [42,43]. The FP-trees were used as a descriptive visualization of baseline medication mechanism co-occurrence patterns. The Apriori algorithm was then used to calculate support values for medication mechanism combinations (min support = 0.1) [44]. Support was defined as the proportion of patients whose first-visit prescriptions contained a given medication mechanism combination. Medication mechanism combinations were ranked according to support values, with a higher rank indicating a more frequently observed baseline prescribing pattern. The Apriori analysis was used only to describe the prevalence of baseline medication mechanism combinations and to define exposure groups. Based on the ranking of support values and the distribution of patient counts across combinations, the top three combinations were retained as separate groups, while all remaining combinations were combined into an “Others” group. This process yielded four mutually exclusive medication mechanism combination groups for subsequent association analyses.

### Network pharmacological analysis

Network pharmacological analysis was conducted to investigate potential medication–target-disease relationships underlying the observed associations. Theraputic targets for medications were searched through the DrugBank (https://www.drugbank.ca/)[38]. Disease targets were collected using the keywords “depression” and “depressive disorder” in the OMIM (https://omim.org/), TTD (http://db.idrblab.net/ttd/) and Genecards (https://www. genecards.org/) [45–47]. The UniProt database (https://www.uniprot.org/) was used to obtain the gene symbols [48]. Cytoscape 3.10.3 was used to construct the medication-target-disease network. Overlapping targets between medications and depression were identified using Venny 2.1.0 (https://bioinfogp.cnb.csic.es/tools/venny/). STRING (https://cn.string-db.org/) was used to analyze protein-protein interactions (PPIs), and to visualize Gene Ontology (GO) and Kyoto Encyclopedia of Genes and Genomes (KEGG) pathway enrichment results [49]. Further topological analysis was performed in Cytoscape 3.10.3 to construct the PPI network, identify the key regulatory proteins, and filter key targets using the Centiscape 2.2 plugin. This analysis was descriptive and hypothesis-generating and was not intended to provide evidence of treatment effectiveness or establish causality.

### Statistical analyses

The characteristics of different trajectory subgroups were compared using the Pearson *χ*^2^ test (or Fisher exact test for small cells) for categorical variables and the analysis of variance test for continuous variables. The associations between the four medication mechanism combination groups and PHQ-9 trajectory subgroups were examined using multinomial logistic regression, with both adjusted and unadjusted ORs calculated. The adjusted model included age, sex, marriage, education, years of working, employment status, smoke, alcohol, and baseline PHQ-9 severity (<10 vs. ≥ 10). A cutoff of 10 was used because it is commonly applied as a clinically meaningful threshold for depression screening and baseline severity stratification. Average predicted probabilities of trajectory membership were estimated from the adjusted multinomial logistic regression model. To further validate the results, the difference between baseline and final SDS standard scores was calculated for each patient. The association between medication mechanism combination groups and SDS score improvement was examined using general linear modeling. Analyses were performed using R version 4.4.2. Analyses were performed using R version 4.4.2. The significance threshold is a 2-sided *P* < 0.05.

## Results

**Fig 1:**
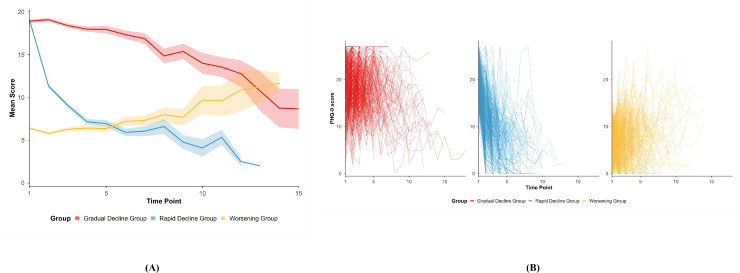
Trajectories of PHQ-9 scores in study population. (A) is subgroup trajectories; (B) is individual trajectories.

After excluding all missing data, the final analytical sample consisted of 1,876 valid respondents (mean [SD] age, 27.60 [11.08] years; 1248 [66.52%] female) (S2 Fig). Participants are classified into three mutually exclusive groups: Rapid Decline Group, Gradual Decline Group, and Worsening Group via GBTM based on their repeated assessed PHQ-9 scores (**Fig 1A**). Rapid Decline Group (n = 562), at 29.96%, shows a rapid decline in mean PHQ-9 scores from the first to the fifth visits, followed by a gradual decline. Gradual Decline Group (n = 577) is almost identical to the Rapid Decline Group in terms of initial PHQ-9 scores, with an overall decreasing trend, but the rate is slower and has not dropped below 5 by the 15th clinical visit. Worsening Group (n = 737) starts with a lower average PHQ-9 scores than the other two groups but shows a consistently upward trend. The individual patient trajectories closely align with the trajectory of their assigned group (**Fig 1B**).

Table 1 presents the univariate comparisons of characteristics among three groups. The results show significant differences in age, marriage, education, years of working, employment status, and alcohol (*P* < 0.001). Sex and smoke are also statistically associated with the trajectory groups (*P* < 0.05).

**Table 1:** Characteristics of participants (N = 1876)^a^.

Characteristics	Overall (N = 1876)	Rapid Decline Group (n = 562)	Gradual Decline Group (n = 577)	Worsening Group (n = 737)	*P* Value^b^
Age, mean (SD), year	27.60 (11.08)	29.06 (11.36)	24.15 (9.91)	29.18 (11.14)	< 0.001
Sex					
Males	628 (33.48%)	184 (32.74%)	164 (28.42%)	280 (37.99%)	0.001
Females	1248 (66.52%)	378 (67.26%)	413 (71.58%)	457 (62.01%)	
Marriage					
Unmarried	1253 (66.79%)	335 (59.61%)	451 (78.16%)	467 (63.36%)	< 0.001
Married	623 (33.21%)	227 (40.39%)	126 (21.84%)	270 (36.64%)	
Education					
High school or below	680 (36.25%)	181 (32.21%)	275 (47.66%)	224 (30.39%)	< 0.001
College and undergraduate	944 (50.32%)	283 (50.36%)	260 (45.06%)	401 (54.41%)	
Master or above	252 (13.43%)	98 (17.44%)	42 (7.28%)	112 (15.20%)	
Years of working, year					
< 2	964 (51.39%)	263 (46.80%)	361 (62.56%)	340 (46.13%)	< 0.001
≥ 2	912 (48.61%)	299 (53.20%)	216 (37.44%)	397 (53.87%)	
Employment status					
Unemployed	1004 (53.52%)	284 (50.53%)	386 (66.90%)	334 (45.32%)	< 0.001
Employed	872 (46.48%)	278 (49.47%)	191 (33.10%)	403 (54,68%)	
Smoke					
No	1584 (84.43%)	465 (82.74%)	474 (82.15%)	645 (87.52%)	0.012
Yes	292 (15.57%)	97 (17.26%)	103 (17.85%)	92 (12.48%)	
Alcohol					
No	1309 (69.78%)	366 (65.12%)	383 (66.38%)	560 (75.98%)	< 0.001
Yes	567 (30.22%)	196 (34.88%)	194 (33.62%)	177 (24.02%)	
Previous psychotropic medication use					
No	1857 (98.99%)	553 (98.40%)	572 (99.13%)	732 (99.32%)	0.236
Yes	19 (1.01%)	9 (1.60%)	5 (0.87%)	5 (0.68%)	

^a^Data are presented as n (%) for categorical variables, and as mean (SD) for continuous variables.

^b^The *P* values were obtained using Pearson *χ*^2^ test for categorical variables and analysis of variance test for continuous variables.

**Fig 2:**
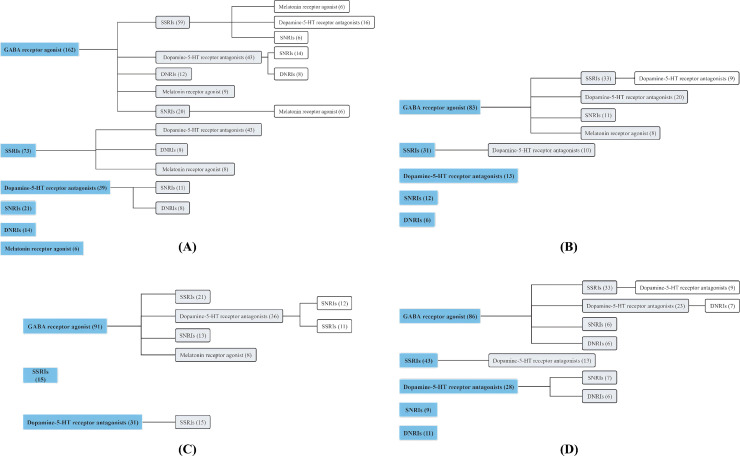
FP trees of different trajectory groups. (A) is total study population; (B) is the Rapid Decline Group; (C) is the Gradual Decline Group. (D) is the Worsening Group. Each node represents a medication mechanism combination identified from first-visit prescriptions, and the numbers in parentheses indicate the count of patients receiving that exact combination at their first visit. The more frequent combinations are positioned toward the left, with branches extending to the right representing co-occurring mechanisms.

Fig 2 iillustrates the FP trees for the overall study population and each PHQ-9 trajectory group.. Co-administration of medications was common in the overall population and across the three trajectory groups. The frequent medication mechanisms were generally consistent across groups. GABAergic agents and SSRIs were the most frequently prescribed. The combination of these two classes of medications was also commonly observed. However, in the Worsening Group, combinations involving DNRIs and dopamine 5-HT receptor antagonists appeared more frequently than in the other groups, whereas combinations of GABAergic agents and melatonin receptor agonists were less frequently observed.

Table 2 shows the main medication mechanism combinations for the Overall, Rapid Decline Group, Gradual Decline Group, and Worsening Group ranked by support value. In the overall population, GABAergic agents (0.568) and SSRIs (0.561) were the most frequent medication mechanisms, which is consistent with the Rapid Decline Group and the Gradual Decline Group.In the Worsening Group, the SSRIs (0.547) remained frequent, but the GABAergic agents (0.440) showed a relatively lower support value.. Compared with the Rapid Decline Group, the Worsening Group showed higher ranks for Dopamine-5-HT receptor antagonists, SSRIs + Dopamine-5-HT receptor antagonists, and SARIs, but lower ranks for GABAergic agents + SSRIs, Melatonin receptor agonists, GABAergic agents + Melatonin receptor agonists, and SNRIs. The Gradual Decline Group, compared with the Rapid Decline Group, exhibits higher ranks for Melatonin receptor agonists, and SSRIs + Dopamine-5-HT receptor antagonists, but lower ranks for GABAergic agents + SSRIs, and SARIs. These support-based patterns were broadly consistent with the medication mechanism co-occurrence structures visually summarized in Fig 2. Based on the support ranking in the overall population and the distribution of patient counts across combinations, the ranked top three were chosen and finalized four completely mutually exclusive groups: GABA, SSRIs, GABA + SSRIs, and Others, comprising 483 (25.75%), 471 (25.11%), 582 (31.02%), and 340 (18.12%) patients, respectively (S2 Table).

**Table 2:** Top 10 medication mechanism combinations in order of support (N = 1876).

Medication mechanism combinations	Overall (N = 1876)		Rapid Decline Group (n = 562)		Gradual Decline Group (n = 577)		Worsening Group (n = 737)	
**Support**	**Rank**	**Support**	**Rank**	**Support**	**Rank**	**Support**	**Rank**
GABAergic agents	0.568	1	0.639	1	0.662	1	0.440	2
SSRIs	0.561	2	0.580	2	0.562	2	0.547	1
GABAergic agents + SSRIs	0.310	3	0.374	3	0.347	4	0.233	4
Melatonin receptor agonists	0.285	4	0.342	4	0.371	3	0.175	5
Dopamine-5-HT receptor antagonists	0.264	5	0.208	6	0.253	6	0.316	3
GABAergic agents + Melatonin receptor agonists	0.196	6	0.247	5	0.274	5	0.096	10
GABAergic agents + Dopamine-5-HT receptor antagonists	0.131	7	0.125	7	0.146	7	0.125	7
SSRIs + Dopamine-5-HT receptor antagonists	0.124	8	0.103	11	0.132	9	0.134	6
SNRIs	0.103	9	0.107	10	0.120	10	0.087	12
SARIs	0.101	10	0.109	9	0.081	14	0.110	8

Note: Support was defined as the proportion of patients whose first-visit prescriptions contained the listed medication mechanism combination. Rank refers to the order from highest to lowest support value within the overall population or each PHQ-9 trajectory group. Support values describe non-mutually exclusive frequent itemsets. The mutually exclusive exposure groups used in subsequent association analyses were derived from these support rankings and are presented in S2 Table.

Among three trajectory groups, there was a significant difference in mechanism combination groups (*P* < 0.001, S2 Table). The association between medication mechanism combination groups and trajectory groups is shown in Table 3. Using the Rapid Decline Group as the reference outcome category and GABA + SSRIs as the reference medication mechanism combination group, no medication mechanism combination was significantly associated with membership in the Gradual Decline Group after adjustment for covariates and baseline PHQ-9 severity (all *P* > 0.05).. For the Worsening Group, after additional adjustment for baseline PHQ-9 severity, SSRIs was associated with 80% increased odds of Worsening trajectory compared with GABA + SSRIs (OR _adj_ = 1.809, 95%CI: 1.196, 2.736). In contrast, the association for the Others group was no longer statistically significant after baseline PHQ-9 adjustment (OR _adj_ = 1.216, 95% CI: 0.757, 1.955). Average predicted probabilities of trajectory group are presented in S3 Table. Compared with GABA+SSRIs (16.6%), patients receiving SSRIs (27.2%) had a higher predicted probability of belonging to the Worsening Group.

**Table 3:** Association of medication mechanism combination groups and trajectory groups.

Medication mechanism combination groups	Gradual Decline Group vs Rapid Decline Group^a^			Worsening Group vs Rapid Decline Group^a^		
**Model 1**	**Model 2**	**Model 3**	**Model 1**	**Model 2**	**Model 3**
GABA + SSRIs^**a**^	Ref.	Ref.	Ref.	Ref.	Ref.	Ref.
GABA	1.283 (0.959, 1.715)	1.238 (0.916, 1.672)	1.226 (0.905, 1.660)	1.246 (0.920, 1.686)	1.259 (0.926, 1.721)	1.127 (0.746, 1.704)
SSRIs	1.122 (0.816, 1.544)	1.013 (0.728, 1.412)	0.975 (0.696, 1.366)	2.431b (1.800, 3.284)	2.413^b^ (1.760, 3.284)	1.809^b^ (1.196, 2.736)
Others	0.857 (0.593, 1.239)	0.743 (0.507, 1.087)	0.685 (0.465, 1.009)	2.554b (1.845, 3.536)	2.476^b^ (1.772, 3.459)	1.216 (0.757, 1.955)

^a^The Rapid Decline Group was the reference outcome category, and the GABA + SSRIs group was the reference medication mechanism combination group.

^b^*P* < 0.05.

Model 1: Unadjusted.

Model 2: Adjusted for age, sex, marriage, education, years of working, employment status, smoke, and alcohol.

Model 3: Model 2 with additional adjustment for baseline PHQ-9 severity.

For the reduction in SDS standard scores, the association between medication mechanism combinations and SDS improvement is shown in S4 Table. After adjustment for demographic and lifestyle covariates and baseline SDS standard score, patients receiving GABA showed significantly less SDS score reduction than those receiving the GABA + SSRIs (β = -1.790, 95% CI: -3.320, -0.258, *P* = 0.022). However, the associations for SSRIs (β = -0.837, 95% CI: -2.410, 0.741, *P* = 0.298) and Others (β = -0.186, 95% CI: -1.940, 1.560, *P* = 0.835) were no longer statistically significant. These results suggest that baseline SDS severity may have partly explained the differences observed in models without baseline SDS adjustment.

Our study incorporated 6 SSRIs (36 potential targets) and 9 GABAergic agents (55 potential targets) (S3 Fig). From three databases, we identified 1104 depression-associated genes and found 33 intersecting targets (S4 Fig). The medication-target-disease network comprised 122 edges and 51 nodes (S5 Fig). PPI analysis yielded a network of intersecting targets and was then collated (S6A Fig). There were 33 nodes and 163 edges, and ten key targets (threshold: closeness = 0.017, betweenness = 30.667, degree = 9.878), more detailed information is shown in S5 Table. GO enrichment revealed 169 biological processes (BPs), 37 cellular components (CC), and 47 molecular functions (MFs). The KEGG pathway primarily involved Neuroactive ligand-receptor interaction, Serotonergic synapse, Calcium signaling, and several addiction and neurotransmission pathways. According to the signals, the top 10 entries of each enrichment result were visualized (S7 Fig).

## Discussion

In this longitudinal study, the use of GABAergic agents and SSRIs combination at the first clinical visit was associated with a more favorable PHQ-9 trajectory group compared with SSRIs monotherapy. After additional adjustment for baseline PHQ-9 severity, the association for SSRIs monotherapy with the Worsening Group remained statistically significant but was attenuated, while the association for other medication mechanism combinations was no longer significant. Predicted probabilities from the adjusted model indicated that the absolute difference in the likelihood of Worsening trajectory membership between SSRIs monotherapy and the GABA+SSRIs combination was approximately 10 percentage points (26.9% versus 16.7%). These findings generate hypotheses for future prospective and randomized studies.

In frequent itemset mining, we found that although the medication mechanism combinations at the first visit were diverse, there was a similarity in the frequent itemsets across groups, which may be associated with physicians’ medication habits. This aligns with prior concerns regarding slow progress and subjectivity in depression treatment [18,50]. In our study, GABAergic agents and SSRIs were frequent prescribed, which is consistent with other studies. SSRIs are the most commonly used first-dose antidepressant [51], and after recognizing the limitations of the monoamine hypothesis, GABAergic-related medications are gradually coming into view [5,52]. This consistency suggests that the medication mechanism combinations identified in our cohort reflect common real-world prescribing patterns. However, frequent itemset mining is descriptive and should not be interpreted as evidence of comparative treatment effectiveness.

The observed association between the initial GABAergic agent + SSRIs and more favorable PHQ-9 trajectories is broadly consistent with prior evidence suggesting that antidepressant combination strategies may be relevant to symptom outcomes. A previous double-blind randomized trial showed that mirtazapine (NaSSA) together with fluoxetine (SSRIs) nearly doubled efficacy compared to fluoxetine alone [53]. Moreover, combining SSRIs with other medication classes (e.g., SNRIs, DNRIs) has shown similar enhancement effects [19]. With the recent approval of GABAergic agents such as brexanolone and zuranolone for postpartum depression, interest in their antidepressant potential has grown [54,55]. However, few studies have explored their synergistic use with SSRIs. Our findings therefore identify this combination as a potentially relevant medication mechanism pattern that warrants further evaluation in prospective studies.

The network pharmacological analysis provided biological context for the observed association between GABAergic agents + SSRIs combination and depressive symptom trajectories. SSRIs elevate extracellular serotonin levels and upregulate GABA concentrations, suggesting a shared pathway of action. Animal studies demonstrate that olanzapine can inhibit GABAergic neurons to disinhibit serotonergic activity, amplifying SSRI effects by 2–3 times [56]. Our PPI analysis revealed ten key targets, including 5-HT receptors (HTR1A, HTR1B, HTR2A, HTR2C) [57–59], serotonin and dopamine transporters (SLC6A4, SLC6A3) [60], GABA-A receptor subunits (GABRA1, GABRG2) [55,61,62], and dopamine receptors (DRD1, DRD2) [63]. These targets are linked to neurotransmitter signaling and synaptic regulation, suggesting possible complementary biological pathways involving SSRIs and GABAergic agents. However, these findings are hypothesis-generating and do not confirm causal mechanisms of symptom improvement.

This exploratory analysis identified four major candidate pathways that may provide biological context for the observed associations: (1) Neural excitation-inhibition balance, with SSRIs enhancing excitatory serotonergic signaling (e.g., GO:0007210) and GABAergic agents suppress hyperexcitability via GABA-A receptor activation (GO:0004890) [62,64–66]; (2) Second messenger signaling, with SSRIs activating the cAMP-PKA-BDNF pathway and both medication classes influencing calcium channel activity [67,68]; (3) Synaptic structure and neurotransmitter modulation, targeting synaptic membranes, GABA-A receptor complexes, and polytransmitter systems (e.g., hsa04080, GO:0007268) [54,64,69]; and (4) Reward circuit regulation, where SSRI-induced serotonin and dopamine release, alongside GABAergic amygdala modulation, may alleviate anhedonia, enriched in addiction-related pathways (e.g., hsa05033, hsa04727, hsa04728) [62,64,70–72]. Notably, vascular regulation pathways (GO:0061028) were implicated, suggesting the need for cerebrovascular assessment in clinical applications. These findings should be interpreted cautiously and require validation in experimental and prospective clinical studies.

This study offers several notable strengths. First, it represents the first to merge GBTM trajectory grouping with frequent itemset mining of medication mechanism combinations, bringing a novel methodological approach. GBTM has been widely used to characterize heterogeneous longitudinal patterns in clinical psychology and medicine [28,73]. Apriori, is a widely-used algorithm in itemset mining now applied innovatively to explore initial-visit medication mechanisms [29,31,32]. Second, by analyzing medications at the mechanism-combination level, this study better reflects real-world psychiatric prescribing practice. Patients often receive multiple medications with different pharmacological mechanisms. Third, the findings were assessed from multiple complementary dimensions. PHQ-9 trajectory modeling captured longitudinal symptom patterns, while changes in SDS standard scores provided a supplementary measure of symptom change. Although the SDS analysis was not fully consistent with the PHQ-9 trajectory findings after baseline SDS adjustment, it provided an additional perspective on symptom change and highlighted the importance of accounting for baseline symptom severity. Finally, network pharmacological analysis provided biological plausibility for the observed associations by linking the identified medication mechanisms with depression-related targets and pathways. Overall, these strengths provide a methodological basis for future studies on medication mechanism combinations in depression treatment, and help inform future mechanistic and prospective studies.

The present study has some limitations. First, because this study was based on a retrospective observational cohort derived from routine EHR data, the observed associations cannot establish causal relationships or comparative treatment effectiveness. Although we adjusted for available covariates and baseline PHQ-9 severity and used SDS score change as a supplementary outcome, first-visit medication selection was not randomized; therefore, confounding by indication and residual confounding cannot be excluded. Unmeasured clinical factors such as symptom profile, anxiety, insomnia, prior treatment history, and clinician preference may still have influenced the results. In addition, because GBTM required at least two PHQ-9 assessments, patients with fewer assessments were excluded, which may have introduced selection bias. PHQ-9 trajectory groups were data-driven constructs rather than directly observed clinical endpoints, which may limit their clinical interchangeability. Second, medications were classified according to their main pharmacological mechanisms. Although this approach facilitated the analysis of complex prescribing patterns, it may not fully capture multi-target effects, medication dosage, regimen changes, treatment duration, or adherence. Because medication exposure was defined based on first-visit prescriptions, subsequent treatment switching, augmentation, or discontinuation may have led to exposure misclassification. Future studies could incorporate more granular and time-varying medication exposure data. Third, the network pharmacological findings were based on existing databases and network-based inference, and should be interpreted as biological context rather than confirmed mechanisms. Future prospective studies with more detailed clinical and time-varying treatment information are needed.

## Conclusion

In this study, utilization of GABAergic agents and SSRIs combination at the first clinical visit was associated with a more favorable PHQ-9 trajectory group compared with SSRIs monotherapy.. This finding may inform future prospective and mechanistic studies on medication mechanism combinations in depression treatment..

## Supporting information

S1 Fig Workflow of this study. (PDF)

S2 Fig Flowchart of the Sample Inclusion and Exclusion. (PDF)

S3 Fig Target networks of GABA + SSRIs combination. (A) shows targets of GABAergic agents; (B) shows targets of SSRIs. The diamond-shaped blocks represent the medication mechanism. The middle layer rectangular blocks represent the medications included in this isochromatic mechanism. The outermost rectangular block represents the relevant targets.
(PDF)

S4 Fig Venny map of GABA + SSRIs combination targets and depressive disorder-related targets and their overlapping. (PDF)

S5 Fig Network diagram of target-active ingredient disease for the anti-depression efficacy of GABA + SSRIs combination. The red diamond-shaped block represents depressive disorder. The blue elliptic blocks represent the depressive disorder-related targets that interact with the GABA + SSRIs combination. The green elliptic blocks represent the medications included in the GABA + SSRIs combination. The yellow diamond-shaped blocks represent GABAergic agents and SSRIs.
(PDF)

S6 Fig The PPI network of targets between GABA + SSRIs combination and depressive disorder. (A) is the PPI network constructed by STRING; (B) is the collated PPI network by Cytoscape. The larger the area of the circle, the higher the degree values. The color of the node’s transitions from blue to red, indicating a higher ranking among gene.
(PDF)

S7 Fig GO functional analysis and KEGG pathway enrichment analyses. (A) shows the Biological Process (Gene Ontology) enrichment analysis results; (B) shows the Cellular Components (Gene Ontology) enrichment analysis results; (C) shows the Molecular Functions (Gene Ontology) enrichment analysis results; (D) shows the KEGG enrichment analysis results. These dot plots illustrate the top 10 enriched terms/pathways in different categories. The x-axis represents the signal value, and the y-axis lists the specific biological processes. The size of each dot indicates the number of genes annotated to the corresponding biological process, with larger dots representing a higher gene count. The color of the dots corresponds to the FDR (False Discovery Rate) value, ranging from blue (lower FDR) to green (higher FDR).
(PDF)

S1 Table Model evaluation metrics of GBTM with different number of groups and polynomial degrees. (PDF)

S2 Table Characteristics of medication mechanism combination groups in trajectory groups (N = 1876). (PDF)

S3 Table Predicted probabilities of trajectory group by medication mechanism combination group. (PDF)

S4 Table Association between the main medication mechanism combinations and reduction in SDS scores. (PDF)

S5 Table Basic information of key targets. (PDF)

S1 File STROBE checklist. The STROBE checklist is licensed under the Creative Commons Attribution 4.0 International License. Source: STROBE Statement, https://www.strobe-statement.org/.
(PDF)
