# Supplementary material for: Association between the combination of GABAergic agents and SSRIs at the first clinical visit and depressive symptom trajectories: A study using group-based trajectory modeling and Apriori algorithm
Source: PLOS Ment Health. 2026 Jul 14;3(7):e0000544. doi: 10.1371/journal.pmen.0000544 (PMC13367733; doi:10.1371/journal.pmen.0000544)
Supplement: S1 Fig — (PDF) [file pmen.0000544.s001.pdf]

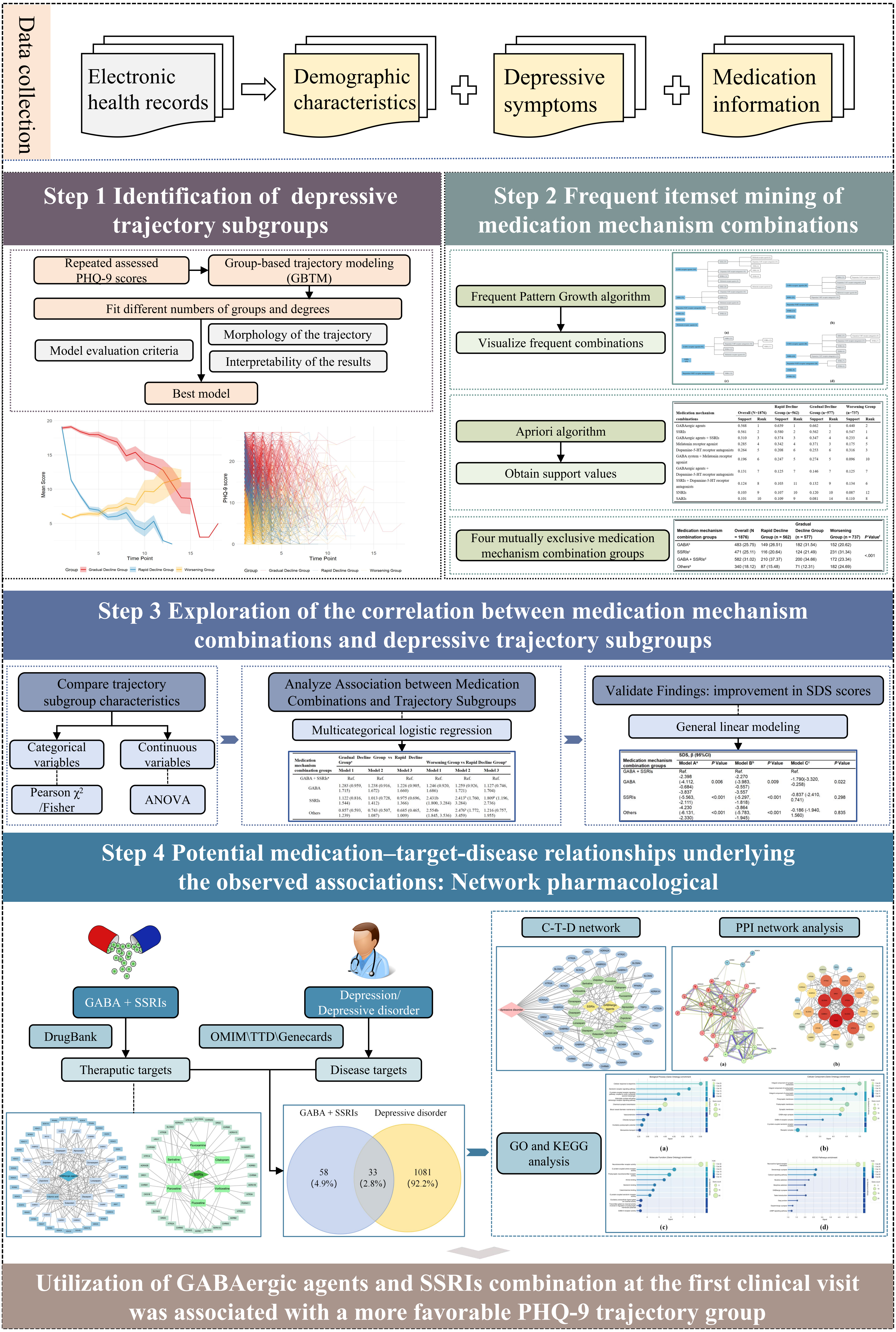

Data collection
Electronic health records
Demographic characteristics
Depressive symptoms
Medication information
Step 1 Identification of depressive trajectory subgroups
Repeated assessed PHQ-9 scores
Group-based trajectory modeling (GBTM)
Fit different numbers of groups and degrees
Morphology of the trajectory
Model evaluation criteria
Interpretability of the results
Best model
Mean Score
Time Point
PHQ-9 score
Group
Gradual Decline Group
Rapid Decline Group
Worsening Group
Step 2 Frequent itemset mining of medication mechanism combinations
Frequent Pattern Growth algorithm
Visualize frequent combinations
Apriori algorithm
Obtain support values
Four mutually exclusive medication mechanism combination groups
Step 3 Exploration of the correlation between medication mechanism combinations and depressive trajectory subgroups
Compare trajectory subgroup characteristics
Categorical variables
Continuous variables
Pearson χ2 /Fisher
ANOVA
Analyze Association between Medication Combinations and Trajectory Subgroups
Multicategorical logistic regression
Validate Findings: improvement in SDS scores
General linear modeling
Step 4 Potential medication–target-disease relationships underlying the observed associations: Network pharmacological
GABA + SSRIs
Depression/ Depressive disorder
DrugBank
OMIM\TTD\Genecards
Theraputic targets
Disease targets
GABA + SSRIs
Depressive disorder
58 (4.9%)
33 (2.8%)
1081 (92.2%)
C-T-D network
PPI network analysis
GO and KEGG analysis
Utilization of GABAergic agents and SSRIs combination at the first clinical visit was associated with a more favorable PHQ-9 trajectory group
