# Supplementary material for: Association between the combination of GABAergic agents and SSRIs at the first clinical visit and depressive symptom trajectories: A study using group-based trajectory modeling and Apriori algorithm
Source: PLOS Ment Health. 2026 Jul 14;3(7):e0000544. doi: 10.1371/journal.pmen.0000544 (PMC13367733; doi:10.1371/journal.pmen.0000544)
Supplement: S2 Fig — (PDF) [file pmen.0000544.s002.pdf]

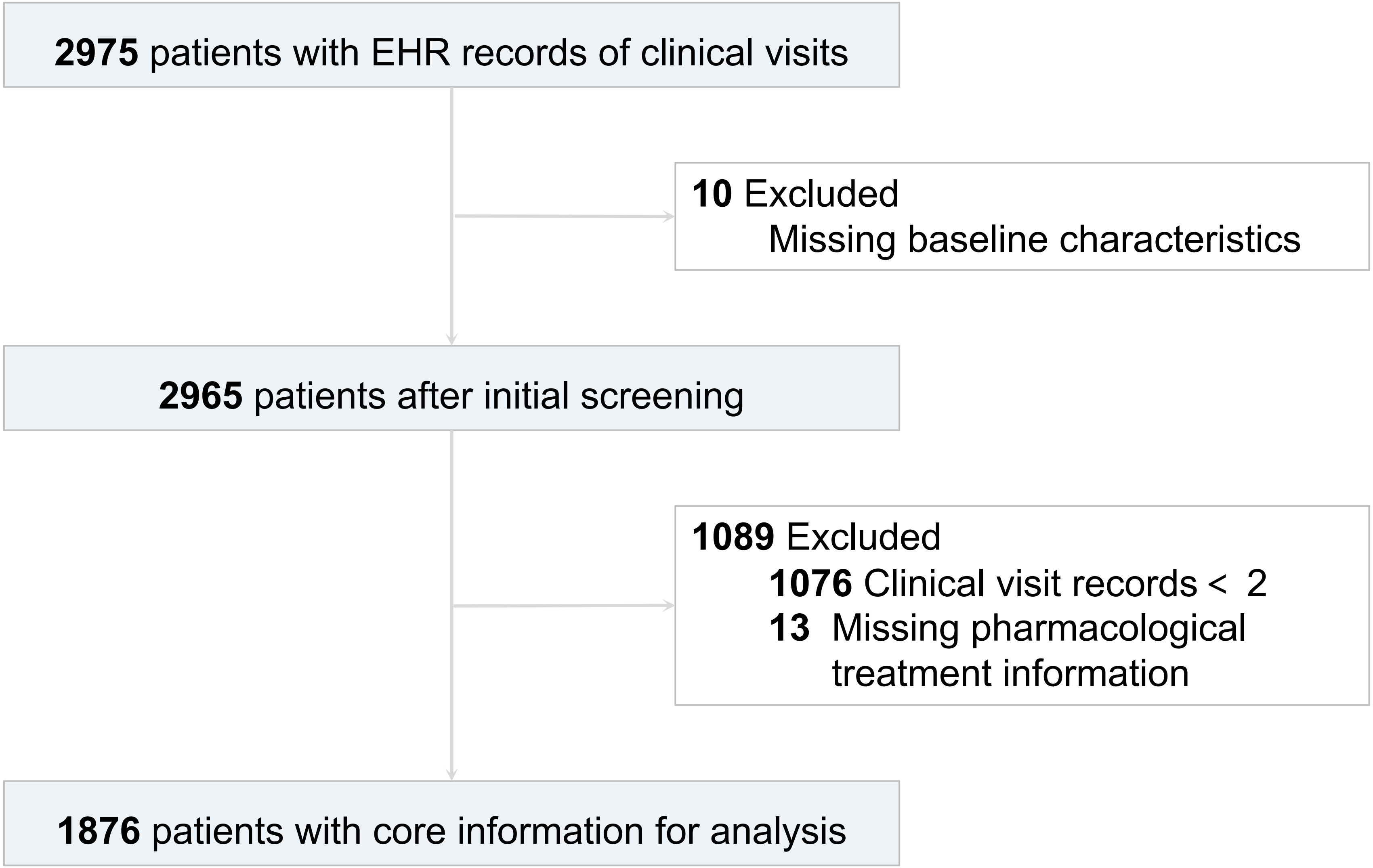
2975 patients with EHR records of clinical visits
10 Excluded
Missing baseline characteristics
2965 patients after initial screening
1089 Excluded
1076 Clinical visit records < 2
13 Missing pharmacological treatment information
1876 patients with core information for analysis
