# Supplementary figures and images for: Association between the combination of GABAergic agents and SSRIs at the first clinical visit and depressive symptom trajectories: A study using group-based trajectory modeling and Apriori algorithm
Source: PLOS Ment Health. 2026 Jul 14;3(7):e0000544. doi: 10.1371/journal.pmen.0000544 (PMC13367733; doi:10.1371/journal.pmen.0000544)

**S3 Fig**

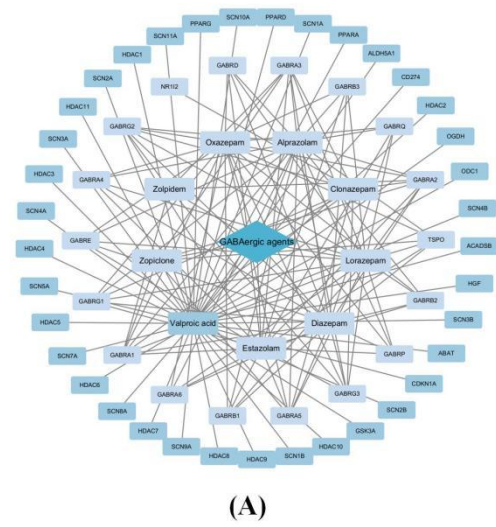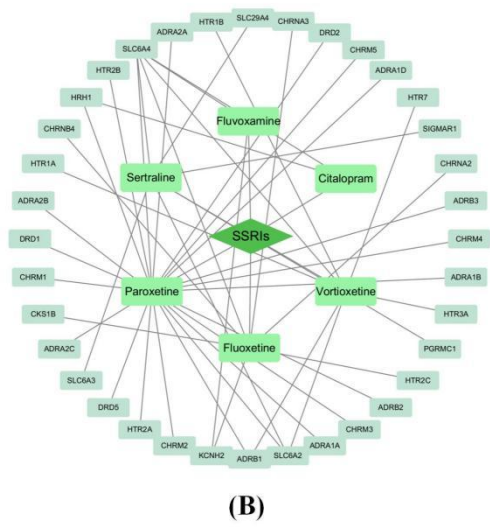

Supplement: S3 Fig — (A) shows targets of GABAergic agents; (B) shows targets of SSRIs. The diamond-shaped blocks represent the medication mechanism. The middle layer rectangular blocks represent the medications included in this isochromatic mechanism. The outermost rectangular block represents the relevant targets. (PDF) [file pmen.0000544.s003.pdf]

**S4 Fig**

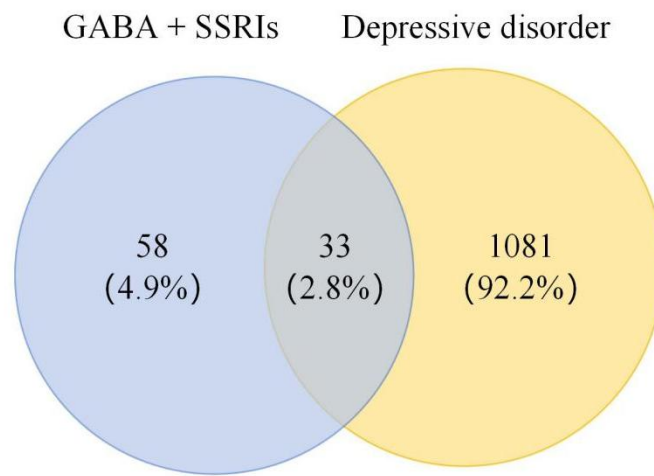

Supplement: S4 Fig — (PDF) [file pmen.0000544.s004.pdf]

**S5 Fig**

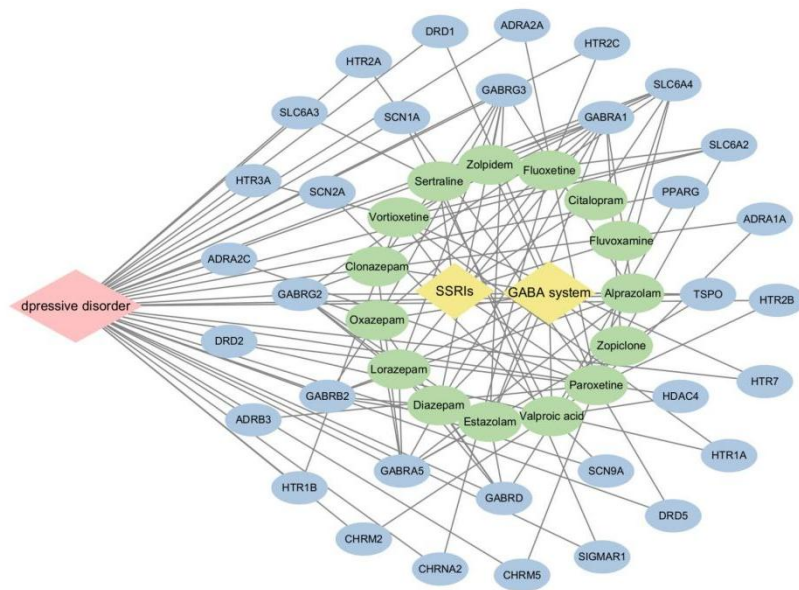

Supplement: S5 Fig — The red diamond-shaped block represents depressive disorder. The blue elliptic blocks represent the depressive disorder-related targets that interact with the GABA + SSRIs combination. The green elliptic blocks represent the medications included in the GABA + SSRIs combination. The yellow diamond-shaped blocks represent GABAergic agents and SSRIs. (PDF) [file pmen.0000544.s005.pdf]

**S6 Fig**

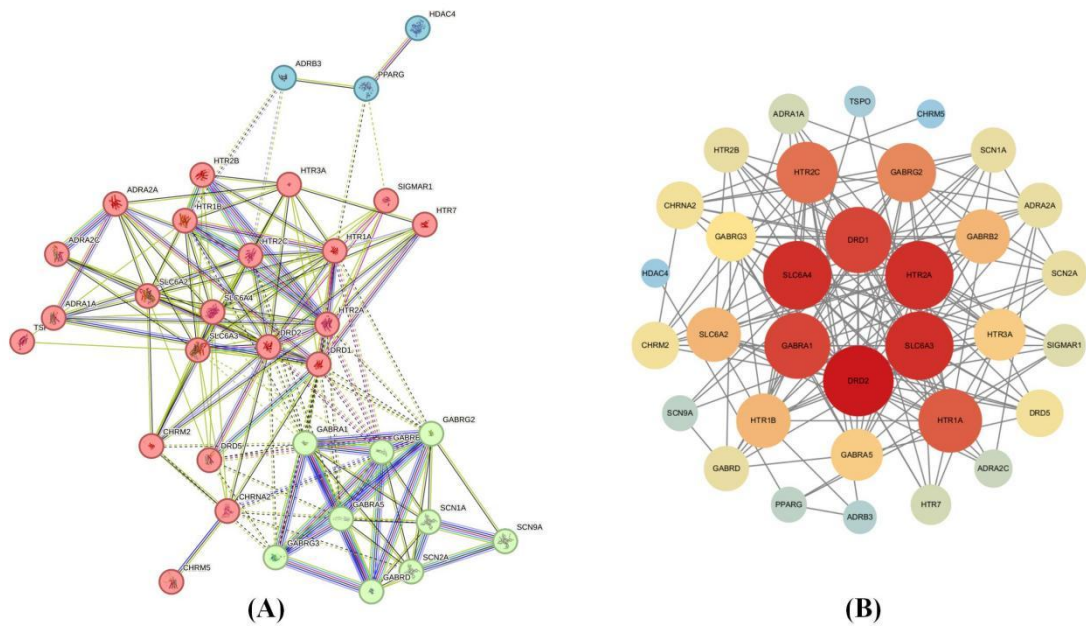

Supplement: S6 Fig — (A) is the PPI network constructed by STRING; (B) is the collated PPI network by Cytoscape. The larger the area of the circle, the higher the degree values. The color of the node’s transitions from blue to red, indicating a higher ranking among gene. (PDF) [file pmen.0000544.s006.pdf]

S7 Fig

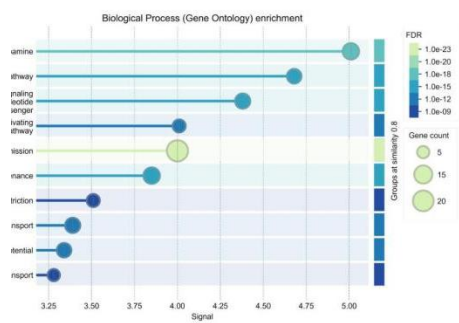

(A)

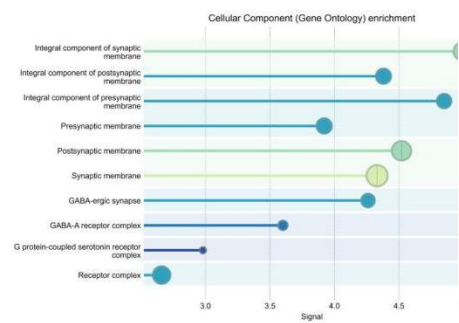

(B)

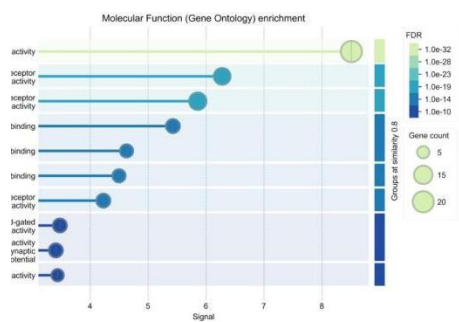

(C)

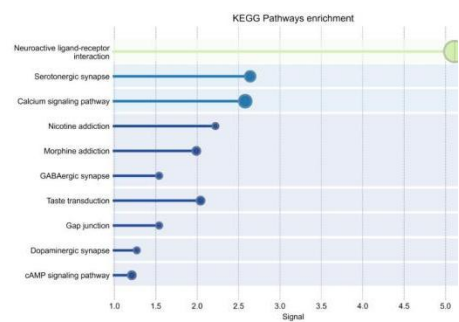

(D)

Supplement: S7 Fig — (A) shows the Biological Process (Gene Ontology) enrichment analysis results; (B) shows the Cellular Components (Gene Ontology) enrichment analysis results; (C) shows the Molecular Functions (Gene Ontology) enrichment analysis results; (D) shows the KEGG enrichment analysis results. These dot plots illustrate the top 10 enriched terms/pathways in different categories. The x-axis represents the signal value, and the y-axis lists the specific biological processes. The size of each dot indicates the number of genes annotated to the corresponding biological process, with larger dots representing a higher gene count. The color of the dots corresponds to the FDR (False Discovery Rate) value, ranging from blue (lower FDR) to green (higher FDR). (PDF) [file pmen.0000544.s007.pdf]
