# Supplementary material for: Association between the combination of GABAergic agents and SSRIs at the first clinical visit and depressive symptom trajectories: A study using group-based trajectory modeling and Apriori algorithm
Source: PLOS Ment Health. 2026 Jul 14;3(7):e0000544. doi: 10.1371/journal.pmen.0000544 (PMC13367733; doi:10.1371/journal.pmen.0000544)
Supplement: S1 Table — (PDF) [file pmen.0000544.s008.pdf]

S1 Table. Model evaluation metrics of GBTM with different number of groups and polynomial degrees.

Groups	Degree	BIC	AIC	ABIC	Entropy	RelativeEntropy	AvePP	OCC
2	1	46265.986	46232.723	46228.267	0.745	1.075	0.923	4279755693068.98
2	2	46173.879	46129.529	46121.073	0.750	1.082	0.925	6228512583890.89
2	3	46122.369	46066.931	46054.475	0.752	1.085	0.926	5907381381263.14
3	1	45654.393	45604.499	45594.043	0.712	0.648	0.864	102304412.13
3	2	45498.107	45431.581	45415.125	0.723	0.658	0.871	3338711464.20
**3***	**3***	**45447.417**	**45364.260**	**45341.804**	**0.724**	**0.659**	**0.871**	**3848346631.78**
4	1	45507.607	45441.081	45424.625	0.615	0.444	0.764	140704170.06
4	2	45409.649	45320.948	45296.492	0.622	0.449	0.765	76907594984.04
4	3	45348.459	45237.582	45205.126	0.679	0.490	0.805	2786833048.25
5	1	45400.063	45316.906	45294.449	0.597	0.371	0.714	2580218010.83
5	2	45313.651	45202.775	45170.319	0.609	0.378	0.725	14581769676.10
5	3	45203.282	45064.687	45022.231	0.638	0.397	0.750	158941238046.89
6	1	45330.901	45231.112	45202.656	0.594	0.331	0.692	2038589296.68
6	2	45125.721	44992.670	44952.214	0.624	0.348	0.719	105960958122.99
6	3	45074.499	44908.185	44855.729	0.644	0.360	0.739	119205922391.24
7	1	45284.447	45168.027	45133.571	0.567	0.291	0.647	287139522.46
7	2	45078.336	44923.110	44874.654	0.600	0.308	0.679	48655477642.71
7	3	45023.602	44829.569	44767.112	0.636	0.327	0.709	596030284842.27

All models converged.

Abbreviation: BIC = Bayesian Information Criterion; AIC = Akaike Information Criterion; ABIC = sample-size adjusted BIC; Entropy = classification entropy (values closer to 1 indicate better classification); RelativeEntropy = relative entropy; AvePP = average posterior probability (values >0.7 suggest good assignment); OCC = odds of correct classification.

*The optimal model (3-group cubic, marked with *) was selected based on a combination of lower BIC, higher entropy and AvePP, meaningful trajectory shapes, and substantive interpretability.
