# Supplementary material for: Association between the combination of GABAergic agents and SSRIs at the first clinical visit and depressive symptom trajectories: A study using group-based trajectory modeling and Apriori algorithm
Source: PLOS Ment Health. 2026 Jul 14;3(7):e0000544. doi: 10.1371/journal.pmen.0000544 (PMC13367733; doi:10.1371/journal.pmen.0000544)
Supplement: S2 Table — (PDF) [file pmen.0000544.s009.pdf]

S2 Table. Characteristics of medication mechanism combination groups in trajectory groups (N = 1876)[a].

Medication mechanism combination groups	Overall (N = 1876)	Rapid Decline Group (n = 562)	Gradual Decline Group (n = 577)	Worsening Group (n = 737)	*P* Value[f]
GABA[b]	483 (25.75)	149 (26.51)	182 (31.54)	152 (20.62)	<0.001
SSRIs[c]	471 (25.11)	116 (20.64)	124 (21.49)	231 (31.34)	
GABA + SSRIs[d]	582 (31.02)	210 (37.37)	200 (34.66)	172 (23.34)	
Others[e]	340 (18.12)	87 (15.48)	71 (12.31)	182 (24.69)	

[a]Data are presented as number (percentage) of patients unless otherwise indicated.
[b]With GABAergic agents, excluding SSRIs.
[c]With SSRIs, excluding GABAergic agents.
[d]With GABAergic agents and SSRIs.
[e]Excluding the other three groups, neither GABAergic agents nor SSRIs.
[f]The *P* values were obtained using Pearson χ^2 test.
