# Supplementary material for: Association between the combination of GABAergic agents and SSRIs at the first clinical visit and depressive symptom trajectories: A study using group-based trajectory modeling and Apriori algorithm
Source: PLOS Ment Health. 2026 Jul 14;3(7):e0000544. doi: 10.1371/journal.pmen.0000544 (PMC13367733; doi:10.1371/journal.pmen.0000544)
Supplement: S3 Table — (PDF) [file pmen.0000544.s010.pdf]

S3 Table. Predicted probabilities of trajectory group by medication mechanism combination group.

Medication group	Rapid Decline Group (n = 562)	Gradual Decline Group (n = 577)	Worsening Group (n = 737)
GABA + SSRIs[a]	0.3770	0.4548	0.1674
GABA[b]	0.3362	0.4959	0.1679
SSRIs[c]	0.3362	0.3945	0.2693
Others[d]	0.4232	0.3488	0.2280

[a]With GABAergic agents and SSRIs.
[b]With GABAergic agents, excluding SSRIs.
[c]With SSRIs, excluding GABAergic agents.
[de]Excluding the other three groups, neither GABAergic agents nor SSRIs.
