# Supplementary material for: Association between the combination of GABAergic agents and SSRIs at the first clinical visit and depressive symptom trajectories: A study using group-based trajectory modeling and Apriori algorithm
Source: PLOS Ment Health. 2026 Jul 14;3(7):e0000544. doi: 10.1371/journal.pmen.0000544 (PMC13367733; doi:10.1371/journal.pmen.0000544)
Supplement: S4 Table — (PDF) [file pmen.0000544.s011.pdf]

S4 Table. Association between the main medication mechanism combinations and reduction in SDS scores.

Medication mechanism combination groups	SDS, β (95%CI)					
	Model A[a]	*P* Value	Model B[b]	*P* Value	Model C[c]	*P* Value
GABA + SSRIs	Ref.		Ref.		Ref.	
GABA	-2.398 (-4.112, -0.684)	0.006	-2.270 (-3.983, -0.557)	0.009	-1.790(-3.320, -0.258)	0.022
SSRIs	-3.837 (-5.563, -2.111)	<0.001	-3.557 (-5.297, -1.818)	<0.001	-0.837 (-2.410, 0.741)	0.298
Others	-4.230 (-6.131, -2.330)	<0.001	-3.864 (-5.783, -1.945)	<0.001	-0.186 (-1.940, 1.560)	0.835

Abbreviation: β, regression coefficient, SDS, self-rating depression scale standard scores.
[a]Unadjusted model.
[b]Adjustment variables: age, sex, marriage, education, years of working, employment status, smoke, and alcohol.
[c]Adjustment variables: Model 2 with additional adjustment for baseline SDS score.
