# Supplementary material for: Association between the combination of GABAergic agents and SSRIs at the first clinical visit and depressive symptom trajectories: A study using group-based trajectory modeling and Apriori algorithm
Source: PLOS Ment Health. 2026 Jul 14;3(7):e0000544. doi: 10.1371/journal.pmen.0000544 (PMC13367733; doi:10.1371/journal.pmen.0000544)
Supplement: S5 Table — (PDF) [file pmen.0000544.s012.pdf]

S5 Table. Basic information of key targets.

No.	Key targets	Closeness Centrality	Betweenness Centrality	Degree Centrality
1	HTR1A	0.0204	108.943	16
2	GABRG2	0.0189	72.526	14
3	GABRA1	0.0204	75.706	17
4	HTR1B	0.0182	41.540	12
5	DRD2	0.0217	70.889	19
6	SLC6A3	0.0208	90.678	18
7	HTR2A	0.0208	61.060	18
8	DRD1	0.0204	71.250	17
9	HTR2C	0.0192	55.583	15
10	SLC6A4	0.0208	60.587	18
