# Supplementary material for: Association between the combination of GABAergic agents and SSRIs at the first clinical visit and depressive symptom trajectories: A study using group-based trajectory modeling and Apriori algorithm
Source: PLOS Ment Health. 2026 Jul 14;3(7):e0000544. doi: 10.1371/journal.pmen.0000544 (PMC13367733; doi:10.1371/journal.pmen.0000544)
Supplement: S1 File — The STROBE checklist is licensed under the Creative Commons Attribution 4.0 International License. Source: STROBE Statement, https://www.strobe-statement.org/. (PDF) [file pmen.0000544.s013.pdf]

STROBE Statement—checklist of items that should be included in reports of observational studies

	Item No.	Recommendation	Page No.	Relevant text from manuscript
**Title and abstract**	1	(*a*) Indicate the study's design with a commonly used term in the title or the abstract	1\3	A Study Using Group-Based Trajectory Modeling and Apriori Algorithm,Longitudinal analysis
		(*b*) Provide in the abstract an informative and balanced summary of what was done and what was found	3-4	Methods\Results\Conclusion
**Introduction**				
Background/rationale	2	Explain the scientific background and rationale for the investigation being reported	5-6	Depression ranks...hypothesis-driven research.
Objectives	3	State specific objectives, including any prespecified hypotheses		This research represents...in treating depression.
**Methods**				
Study design	4	Present key elements of study design early in the paper	6	This research represents...in treating depression.
Setting	5	Describe the setting, locations, and relevant dates, including periods of recruitment, exposure, follow-up, and data collection	7	For this longitudinal study... from the outpatient.
Participants	6	(*a*) *Cohort study*—Give the eligibility criteria, and the sources and methods of selection of participants. Describe methods of follow-up<br>*Case-control study*—Give the eligibility criteria, and the sources and methods of case ascertainment and control selection. Give the rationale for the choice of cases and controls<br>*Cross-sectional study*—Give the eligibility criteria, and the sources and methods of selection of participants	7	For this longitudinal study... from the outpatient.
		(*b*) *Cohort study*—For matched studies, give matching criteria and number of exposed and unexposed<br>*Case-control study*—For matched studies, give matching criteria and the number of controls per case		
Variables	7	Clearly define all outcomes, exposures, predictors, potential confounders, and effect modifiers. Give diagnostic criteria, if applicable	8-10	Patient Health Questionnaire-9 (PHQ-9)...Centiscape 2.2 plugin.
Data sources/ measurement	8*	For each variable of interest, give sources of data and details of methods of assessment (measurement). Describe comparability of assessment methods if there is more than one group	8-10	Patient Health Questionnaire-9 (PHQ-9)...Centiscape 2.2 plugin.
Bias	9	Describe any efforts to address potential sources of bias	11	To further validate the results...linear modeling.
Study size	10	Explain how the study size was arrived at	7	we utilized electronic health records (EHR) data...

Continued on next page

Quantitative variables	11	Explain how quantitative variables were handled in the analyses. If applicable, describe which groupings were chosen and why	10	The characteristics of..variables
Statistical methods	12	(*a*) Describe all statistical methods, including those used to control for confounding	10-11	Statistical analyses...2-sided $P < 0.05$.
		(*b*) Describe any methods used to examine subgroups and interactions		
		(*c*) Explain how missing data were addressed		
		(*d*) *Cohort study*—If applicable, explain how loss to follow-up was addressed<br>*Case-control study*—If applicable, explain how matching of cases and controls was addressed<br>*Cross-sectional study*—If applicable, describe analytical methods taking account of sampling strategy		
		(*e*) Describe any sensitivity analyses		
**Results**				
Participants	13*	(a) Report numbers of individuals at each stage of study—eg numbers potentially eligible, examined for eligibility, confirmed eligible, included in the study, completing follow-up, and analysed	11\S2 Fig	After excluding...(S2 Fig).
		(b) Give reasons for non-participation at each stage		
		(c) Consider use of a flow diagram		
Descriptive data	14*	(a) Give characteristics of study participants (eg demographic, clinical, social) and information on exposures and potential confounders	11-12\ Table 1	After excluding....<br>Table 1 presents the...($P < 0.05$).
		(b) Indicate number of participants with missing data for each variable of interest		
		(c) *Cohort study*—Summarise follow-up time (eg, average and total amount)		
Outcome data	15*	*Cohort study*—Report numbers of outcome events or summary measures over time	11-15	Participants are classified...<br>(S1 Table).
		Case-control study—Report numbers in each exposure category, or summary measures of exposure		
		Cross-sectional study—Report numbers of outcome events or summary measures		
Main results	16	(*a*) Give unadjusted estimates and, if applicable, confounder-adjusted estimates and their precision (eg, 95% confidence interval). Make clear which confounders were adjusted for and why they were included	15-16	Among three trajectory groups...<br>with both GABA + SSRIs.
		(*b*) Report category boundaries when continuous variables were categorized		
		(*c*) If relevant, consider translating estimates of relative risk into absolute risk for a meaningful time period		

Continued on next page

Other analyses	17	Report other analyses done—eg analyses of subgroups and interactions, and sensitivity analyses	16-17	For SDS standard scores reduction... Our study incorporated 6 SSRIs...
**Discussion**				
Key results	18	Summarise key results with reference to study objectives	17	In this longitudinal study...depression treatment.
Limitations	19	Discuss limitations of the study, taking into account sources of potential bias or imprecision. Discuss both direction and magnitude of any potential bias	20-21	The present study has some limitations.
Interpretation	20	Give a cautious overall interpretation of results considering objectives, limitations, multiplicity of analyses, results from similar studies, and other relevant evidence	17-19	In frequent itemset mining,...in clinical applications.
Generalisability	21	Discuss the generalisability (external validity) of the study results	20	The present study offers...therapeutic needs.
**Other information**				
Funding	22	Give the source of funding and the role of the funders for the present study and, if applicable, for the original study on which the present article is based	The journal requires that this information not be displayed in the submitted manuscript but instead be submitted through the submission system.	

*Give information separately for cases and controls in case-control studies and, if applicable, for exposed and unexposed groups in cohort and cross-sectional studies.

Note: An Explanation and Elaboration article discusses each checklist item and gives methodological background and published examples of transparent reporting. The STROBE checklist is best used in conjunction with this article (freely available on the Web sites of PLoS Medicine at http://www.plosmedicine.org/, Annals of Internal Medicine at http://www.annals.org/, and Epidemiology at http://www.epidem.com/). Information on the STROBE Initiative is available at www.strobe-statement.org.
